# *Fusobacterium nucleatum* promotes colorectal cancer metastasis through miR-1322/CCL20 axis and M2 polarization

**DOI:** 10.1080/19490976.2021.1980347

**Published:** 2021-10-10

**Authors:** Chaochao Xu, Lina Fan, Yifeng Lin, Weiyi Shen, Yadong Qi, Ying Zhang, Zhehang Chen, Lan Wang, Yanqin Long, Tongyao Hou, Jianmin Si, Shujie Chen

**Affiliations:** a Department of Gastroenterology, Sir Run Run Shaw Hospital, School of Medicine, Zhejiang University, Hangzhou, China; b Department of Gastroenterology, Second Affiliated Hospital of Zhejiang University School of Medicine, Hangzhou, China; c Institute of Gastroenterology, Zhejiang University, Hangzhou, China; d Cancer Center, Zhejiang University, Hangzhou, Zhejiang, China

**Keywords:** Colorectal cancer, *Fusobacterium nucleatum*, macrophage infiltration, CCL20, metastasis

## Abstract

Colorectal cancer (CRC) is one of the most common malignant tumors and is associated with *Fusobacterium nucleatum* (*F. nucleatum, Fn*) infection. In this study, we explored the role of *F. nucleatum* in the CRC metastasis. Our results showed that the abundance of *F. nucleatum* was enriched in the feces and tumors of patients with CRC and tended to increase in stage IV compared to stage I in patients with metastatic CRC. Tumor-derived CCL20 activated by *F. nucleatum* not only increases CRC metastasis, but also participates in the reprograming of the tumor microenvironment. *F. nucleatum* promoted macrophage infiltration through CCL20 activation and simultaneously induced M2 macrophage polarization, enhancing the metastasis of CRC. In addition, we identified using database prediction and luciferase activity hat miR-1322, a candidate regulatory micro-RNA, could bind to CCL20 directly. *F. nucleatum* infection decreased the expression of miR-1322 by activating the NF-κB signaling pathway in CRC cells. In conclusion, *F. nucleatum* promotes CRC metastasis through the miR-1322/CCL20 axis and M2 polarization.

## Introduction

The global incidence of colorectal cancer (CRC) ranks third among that of all malignant tumors, and the fatality rate ranks fourth.^1^ Approximately 50% of CRC patients develop metastases, which are the primary cause of death.^2^ At present, despite various clinical advances in chemotherapy and targeted therapy for patients with metastatic CRC, the 5-year survival rate remains still unsatisfactory,^3,4^ and treatment failure is often associated with metastatic diffusion.^5,6^ Therefore, the mechanism underlying CRC metastasis should be investigated.

Human intestinal flora comprises a wide variety of microorganisms. Metagenomic sequencing, bacterial culturing, sterile mouse breeding, and other research means, have been revealed that various intestinal flora (e.g., *Fusobacterium nucleatum, Bacteroides fragilis*, and *toxigenic Escherichia coli*) are involved in the occurrence and development of CRC. *F. nucleatum*, an invasive, adherent, and pro-inflammatory anaerobe, plays a vital role in the regulation of the gut immune system.^7^

*F. nucleatum* inhibits anti-tumor immunity of gut mucosa by suppressing the function of immune cells including NK cells, tumor-associated neutrophils (TANs), T cells, and macrophage in TME, facilitating the progression of CRC.^8–10^ Chemokines belong to a family of chemotactic cytokines and are associated with multiple tumorigenesis. Chemokines can directly regulate the proliferation, angiogenesis, migration, and chemotherapeutic resistance of cancer cells.^11^ They also play a key role in the progression of cancer by recruiting immune cells in the TME.^12–14^Whether or not *F. nucleatum* influences chemokines and reprograms the TME to promote CRC metastasis remains unclear; therefore, the mechanisms underlying the role of this microorganism on CRC need to be fully elucidated.

In this study, we aimed to determine the relationship between *F. nucleatum* infection and CRC metastasis and its underlying molecular mechanisms. We found that *F. nucleatum* infection promoted CRC metastasis through the miR-1322/CCL20 axis and M2 polarization. Our data offer a new opportunity for targeting chemokines and immune homeostasis for the treatment of CRC metastasis.

## Results

### *F. nucleatum* is more abundant in CRC and increases CRC cell invasion and migration

**Figure 1.**
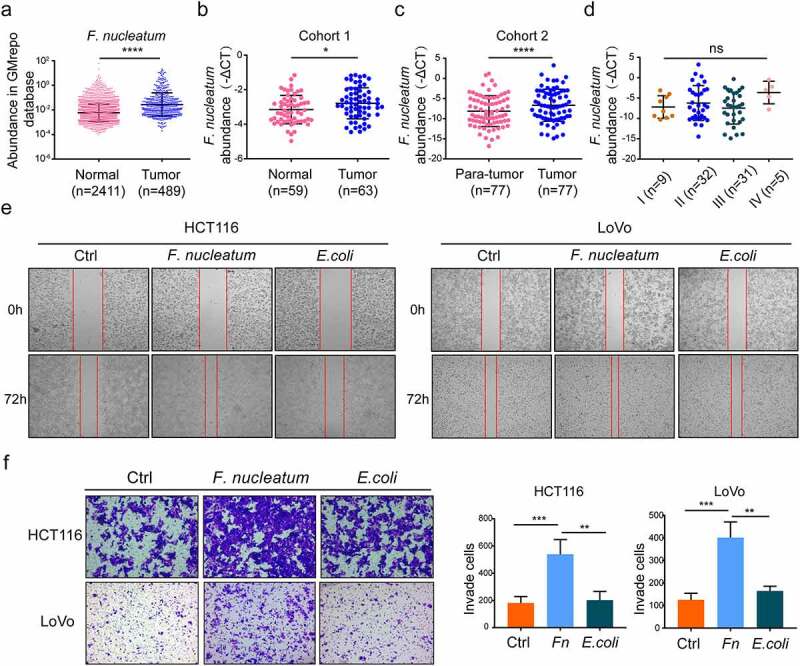
**The *F. nucleatum* was enriched in colorectal cancer and enhanced CRC cells invasion and migration**. (a) The fecal abundance of *F. nucleatum* among the healthy and CRC patients in the GMrepo database (Mann-Whitney test). (b) Fecal bacteria genomic DNA was extracted from the healthy population (n = 59), CRC patients (n = 63) in cohort 1. The abundance of *F. nucleatum* was examined by qPCR. (Cohort 1, Mann-Whitney test). (c) Bacteria genomic DNA was extracted from CRC tissues and adjacent normal mucosa (n = 77) in cohort 2. The abundance of *F. nucleatum* was tested by qPCR. (d) The abundance of *F. nucleatum* among CRC patients with different TNM stage in our tested CRC tissues (One-way ANOVA test). (e, f) After incubated with PBS or *E. coli* or *F. nucleatum*, the mobility of HCT116 and LoVo cells was detected by wound healing assay (e) or transwell assay (f). Every 6 fields were counted for each sample (Each experiment was repeated in triplicate; Student’s t test). Scale bars:100 μm. * *p* < .05, ** *p* < .01, and *** *p* < .001, **** *p* < .0001, ns no significant

We analyzed the abundance of *F. nucleatum* in fecal samples to examine the relationship between *F. nucleatum* and CRC. We found that *F. nucleatum* abundance was significantly increased in fecal samples of CRC patients in the GMrepo database (Figure 1(a)), which was consistent with the results from fecal samples in cohort 1 (Figure 1(b)). In addition, the results of qPCR determined that *F. nucleatum* abundance was significantly higher in the tumor tissues of CRC patients than in the adjacent normal tissues (cohort 2; Figure 1(c)). Although not statistically significant, the abundance of *F. nucleatum* tended to increase in stage IV compared with stage I in patients with metastatic CRC (Figure 1(d)). Moreover, we used Transwell and wound healing assays to assess the mobility of HCT116 and LoVo cells after incubation with *F. nucleatum* or *E. coli* for 24 h. We found that *F. nucleatum* infection significantly increased CRC cell migration and invasion compared with *E. coli* or PBS (phosphate buffer saline) (Figure 1(e-f)). These results suggest that *F. nucleatum* plays a vital role in CRC metastasis.

### *F. nucleatum* promotes the metastasis of CRC cells in a CCL20 dependent manner

**Figure 2.**
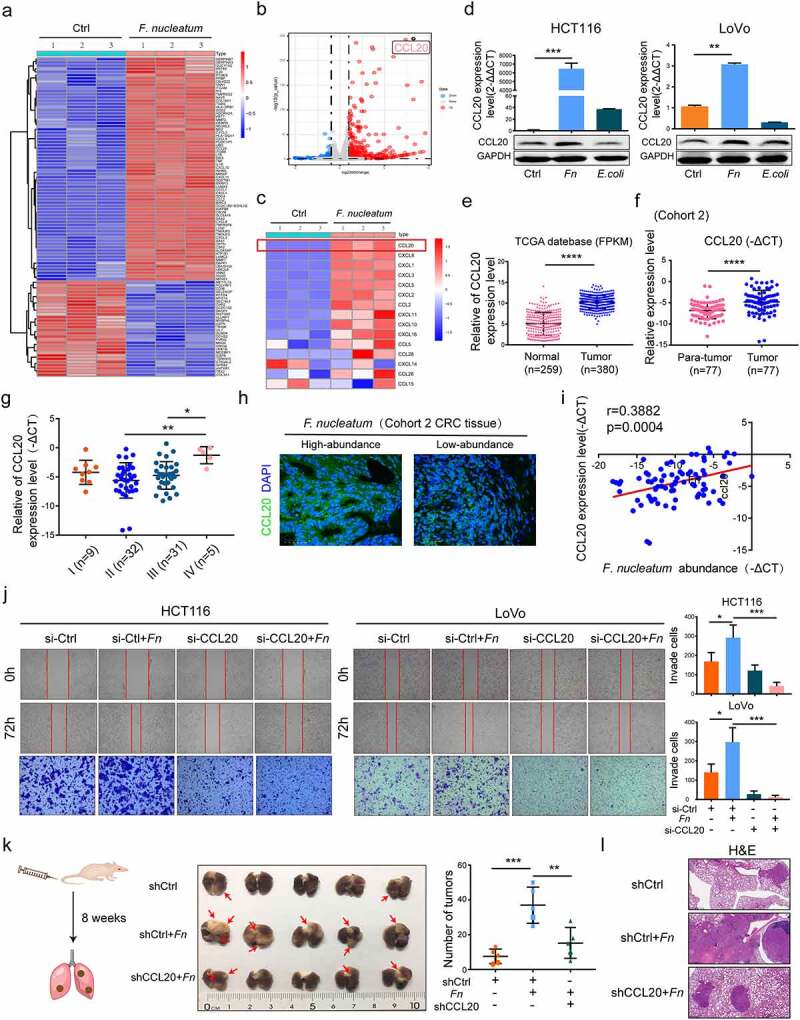
***F. nucleatum* promoted metastasis of CRC cells in a CCL20 dependent manner**. (a, b) Heat map(a) and Vo**l**cano map(b) representing the differential gene expression patterns between *F. nucleatum*-treated and PBS-treated LoVo cell by Microarrays (n = 3 per group, fold change>2, logCPM>2, FDR<0.05). (c) The expression of CCL20 among all chemokines in LoVo cell after incubated with *F. nucleatum* or PBS was represented by heat map (n = 3 per group, fold change>2, logCPM>2, FDR<0.05). (d)The protein and mRNA expression of CCL20 after *F. nucleatum* or *E. coli* or PBS treated in CRC cells (Student’s t test). (e) Relative expression of CCL20 in TCGA database (Mann-Whitney test). (f)Relative expression of CCL20 in the CRC tissues (cohort 2, n = 77 pairs; Paired Student’s t test). (g) Relative expression of CCL20 among CRC patients with different TNM stage in our tested CRC tissues (One-way ANOVA test). (h) The CCL20 expression was detected by Immunofluorescence in *F. nucleatum* high and low abundance CRC tissues. Scale bars:25 μm. (i) The correlation between CCL20 expression and *F. nucleatum* abundance in our tested CRC tissues (n = 77; spearman correlation analysis). (j) Pretreated with CCL20 siRNA or control siRNA, CRC cells were incubated with *F. nucleatum* or PBS for 24 h, the mobility of CRC cells was detected by transwell and wound healing assays (Each experiment was repeated in triplicate; Student’s t test). Scale bars:100 μm. (k, l) Pretreated with CCL20 shRNA or control shRNA, HCT116 cells were incubated with *F. nucleatum* or PBS for 24 h. Then HCT116 cells were injected into nude mice via tail vein (n = 5 each group). The red arrows indicated metastatic nodules. The number of metastatic lesions (k) (Student’s test test) and the representative images of H&E staining (l) in each group. Scale bars:200 μm. * *p* < .05, ** *p* < .01, and *** *p* < .001, **** *p* < .0001, ns no significant

Upregulation of chemokine expression plays a vital role in the progression of cancer.^11^ To investigate whether chemokines were involved in *F. nucleatum*-induced CRC metastasis, we used an RNA-seq profiles previously developed by our team.^15^ Heatmap and volcano map showed that CCL20 was one of the most differentially upregulated chemokines in *F. nucleatum*-infected LoVo cells compare with PBS treatment (Figure 2(a-c)). To confirm the results of RNA-seq, the CCL20 expression of HCT 116 or LoVo cells was analyzed by qPCR and western blot analysis after incubation with *F. nucleatum, E. coli*, or PBS for 24 h. We observed that the expression of CCL20 was significantly upregulated in *F. nucleatum*-infected cells (Figure 2(d)). Using the Cancer Genome Atlas (TCGA) database, we found that CCL20 expression was upregulated in CRC patients (Figure 2(e)). The expression of CCL20 was also significantly increased in the samples used in our study (cohort 2; Figure 2(f)). Meanwhile, CCL20 mRNA expression tended to increase in stage IV compared with stages II and III in patients with metastatic CRC (Figure 2(g)). Immunofluorescence showed increased expression of CCL20 in CRC tissues with high *F. nucleatum* abundance relative to those with low abundance (Figure 2h). In addition, CCL20 expression was positively associated with the abundance of *F. nucleatum* in the CRC patient tissues used in this study (Figure 2(i)). To further explore the role of CCL20 in CRC metastasis, CCL20 loss-of-function assays were performed. The metastasis-promoting effect induced by *F. nucleatum* was alleviated by CCL20 knockdown in CRC cells (Figure 2(j)). To further assess the role of CCL20 in *F. nucleatum*-mediated CRC metastasis *in vivo*, HCT116 cells transfected with CCL20 short hairpin RNA (shRNA) or control plasmids were incubated with *F. nucleatum* for 24 h and then injected into the tail vein of BALB/C nude mice. *F. nucleatum* infection significantly increased the number of metastatic lesions, while the knockdown of CCL20 significantly reduced *F. nucleatum*-induced CRC lung metastasis (Figure 2(k-l)). Taken together, these data indicate that *F. nucleatum* promotes CRC metastasis by upregulating CCL20 expression *in vitro* and *in vivo*.

### Tumor-derived CCL20 expression induced by *F. nucleatum* increases macrophage infiltration in the TME

**Figure 3.**
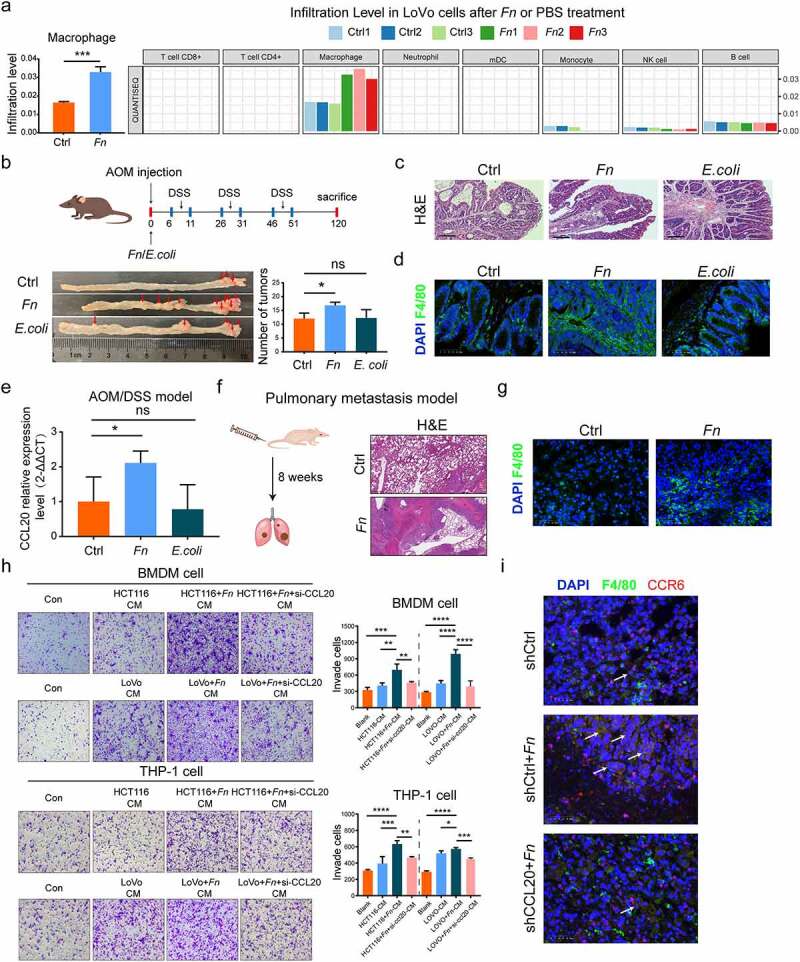
**Tumor-derived CCL20 induced by *F. nucleatum* mediated the macrophage infiltration in the TME**. (a) The immune cells infiltration was analyzed via TIMER 2.0 and quanTIseq based on RNA-seq profiles. (b) Colon tumor number in AOM/DSS model was counted in *F. nucleatum* or PBS or *E. coli* groups (Student’s test; n = 4–8 per group). The red arrows indicated the tumor locations. (c) Representative images of H&E staining in the intestinal tissues in AOM/DSS CRC model after *F. nucleatum* or PBS or *E. coli* treatment. Scale bars:500 μm. (d) The proportion of F4/80^+^ macrophage was detected by immunofluorescence in AOM/DSS CRC model after *F. nucleatum* or PBS or *E. coli* treatment (Student’s t test). Scale bars:25 μm. (e) The CCL20 expression was detected in AOM/DSS model after *F. nucleatum* or PBS or *E. coli* treatment (Student’s t test). (f) Representative images of H&E staining in the pulmonary metastasis model after *F. nucleatum* or PBS treatment. Scale bars:200 μm. (g)The proportion of F4/80^+^ macrophage was detected by immunofluorescence in the pulmonary metastasis model after *F. nucleatum* or PBS treatment. Scale bars:25 μm. (h) After co-cultured with culture medium of CRC cells pretreated by *F. nucleatum* and/or si-RNA CCL20 for 24 h, the mobility of macrophage was detected by transwell assay (Each experiment was repeated in triplicate; Student’s t test). Scale bars:100 μm. (i) Representative images of the F4/80^+^ CCR6^+^ macrophage in the pulmonary metastasis model after control shRNA or control shRNA+ *F. nucleatum* or CCL20 shRNA+ *F. nucleatum* treatment were detected by immunohistochemistry. The white arrows indicated F4/80^+^ CCR6^+^ macrophage. Scale bars:25 μm. * *p* < .05, ** *p* < .01, and *** *p* < .001, **** *p* < .0001, ns no significant

Immune cells in the TME play a vital role against or in promoting tumorigenesis and progression. Recent studies have reported that *F. nucleatum* inhibits anti-tumor immunity of the gut mucosa to facilitate the progression of CRC by regulating immune cells.^8–10^ Immune cell infiltration during *F. nucleatum* infection was systematically analyzed to further explore the interaction between *F. nucleatum* and the host immune system during CRC metastasis. We used an RNA-seq profiles previously developed by our team.^15^ TIMER 2.0 and quanTIseq^16,17^ showed a positive correlation between *F. nucleatum* infection and macrophage infiltration in *F. nucleatum*-treated LoVo cells compared with PBS-treated (Figure 3(a)). An azoxymethane/dextran sodium sulfate (AOM/DSS) model confirmed that *F. nucleatum* enhanced the tumorigenesis of CRC (Figure 3(b)), and this was verified by H&E staining (Figure 3(c)). No statistical difference was found between *E. coli* and the PBS group. The AOM/DSS model showed that *F. nucleatum* infection indeed increased the proportion of F4/80^+^ macrophage as visualized by immunofluorescence (Figure 3(d)), which was consistent with the results of immune cell infiltration analysis (Figure 3(a)), and mRNA CCL20 expression (Figure 3(e)) compared with the PBS treatment in the TME. Moreover, *F. nucleatum* infection significantly increased CRC metastasis relative to the PBS treatment (Figure 3(f)), and simultaneously increased the proportion of F4/80^+^ macrophage as visualized by immunofluorescence in the pulmonary metastasis model (Figure 3(g)).

CCL20, the only known ligand for the receptor CCR6, not only influences cancer progression but also participates in TME reprogramming by controlling immune cells.^18^ We used a Transwell assay to explore the relationship between macrophage infiltration and *F. nucleatum*-induced CCL20 expression *in vitro*. After pretreated with *F. nucleatum* alone or together with CCL20 small interfering RNA (siRNA), the CRC cell conditional medium was collected for macrophage incubation. We found that the mobility of THP-1-derived macrophage and bone marrow-derived macrophages (BMDMs) was increased, and this effect was partially inhibited by downregulating CCL20 expression (Figure 3(h)). In addition, we found that the expression of F4/80^+^ CCR6^+^ macrophage in lung metastasis tissues was significantly decreased in the CCL20 shRNA+ *F. nucleatum* group compared with the shCtl+ *F. nucleatum* group (Figure 3(i)). Our study found that *F. nucleatum* enhances tumor-derived CCL20 expression and recruits F4/80^+^ CCR6^+^ macrophage in the TME.

### *F. nucleatum* promotes M2 macrophage polarization, enhancing CRC metastasis

**Figure 4.**
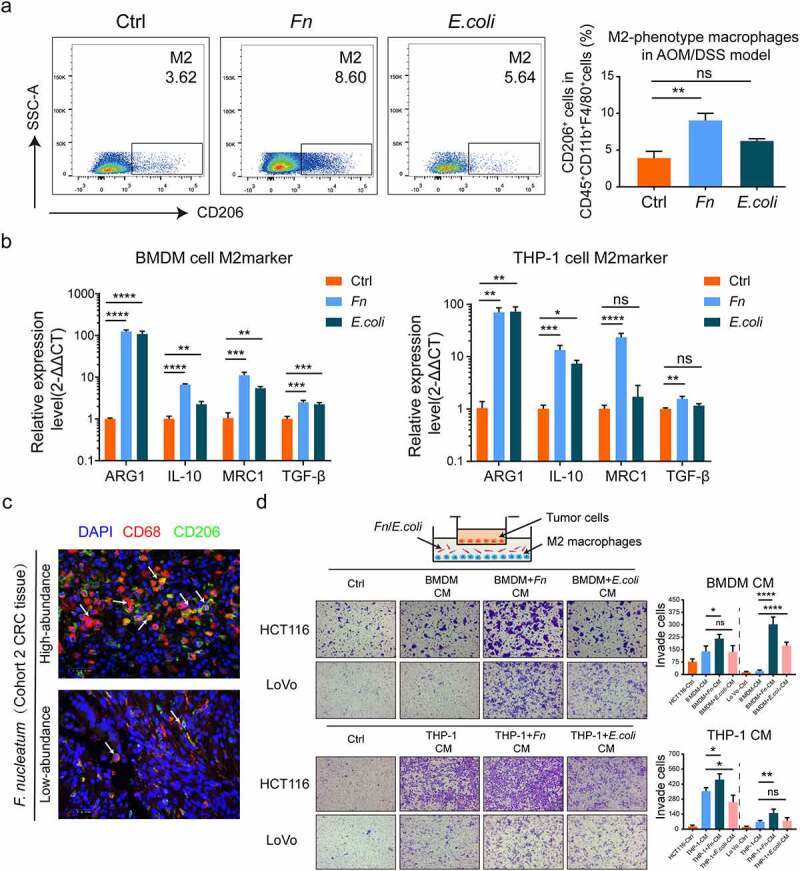
***F. nucleatum* induced the M2 polarization of macrophage to promote CRC metastasis**. (a) Frequencies of M2-phenotype macrophage (CD45^+^ CD11b^+^ F4/80^+^ CD206^+^) from colon lamina propria in AOM/DSS model after *F. nucleatum* or PBS or *E. coli* treatment were tested by multicolor flow cytometry. (Student’s t test). (b)The mRNA expression of M2 markers (ARG1, MRC1, IL-10 and TGF-β) were detected in THP-1-derived macrophage and BMDMs after being infected with *F. nucleatum* or *E. coli* or PBS for 24 h (Student’s t test). (c) Representative images of M2-phenotype macrophage (CD68^+^ CD206^+^) were examined by immunofluorescence in *F. nucleatum* high and low abundance CRC tissues. Scale bars:25 μm. The white arrows indicated CD68^+^ CD206^+^ M2-phenotype macrophage. (d) After co-cultured with culture medium of Macrophage pretreated with *F. nucleatum* or *E. coli* or PBS for 24 h, the mobility of CRC cells was detected by transwell assay (Each experiment was repeated in triplicate; Student’s t test). Scale bars:100 μm. * *p* < .05, ** *p* < .01, and *** *p* < .001, **** *p* < .0001, ns no significant

Macrophage, an essential innate immune cell for maintaining system homeostasis, showed high plasticity and could polarize into M1- or M2-phenotype. M2-phenotype macrophage in the TME play a vital role in promoting tumor progression.^19^ Therefore, to determine whether *F. nucleatum* affects macrophage polarization in the AOM/DSS model, we analyzed M2-phenotype macrophage from colon lamina propria by flow cytometry assays. We found that *F. nucleatum* infection significantly increased M2-phenotype macrophage (CD45^+^ CD11b^+^ F4/80^+^ CD206^+^) proportion compared with the PBS group; there was no significant difference compared to the *E. coli* group (Figure 4(a)). M2-phenotype macrophage markers were identified by qPCR in THP-1-derived macrophage and BMDMs. The expression of M2 markers (ARG1, MRC1, IL-10, and TGF-β) at the mRNA level was upregulated by *F. nucleatum* compared with PBS, but also upregulated in the *E. coli* group (Figure 4(b)). In addition, M2-phenotype macrophage (CD68+ CD206+) were examined by immunofluorescence in human CRC tissues with high or low *F. nucleatum* abundance. The densities of M2-phenotype macrophage (CD68+ CD206+) were higher in CRC tissues with high *F. nucleatum* abundance than in those with low *F. nucleatum* abundance (Figure 4(c)). Moreover, THP-1-derived macrophage and BMDMs infected with *F. nucleatum* significantly enhanced the mobility of HCT116 and LoVo cells *in vitro* compared with those treated with *E. coli* or PBS (Figure 4(d)). Together, our results reveal important aspects of the impact of *F. nucleatum* on macrophage infiltration and the simultaneous induction of M2 macrophage polarization, enhancing CRC metastasis.

### Tumor-derived CCL20 expression by *F. nucleatum* is regulated by miRNA-1322

**Figure 5.**
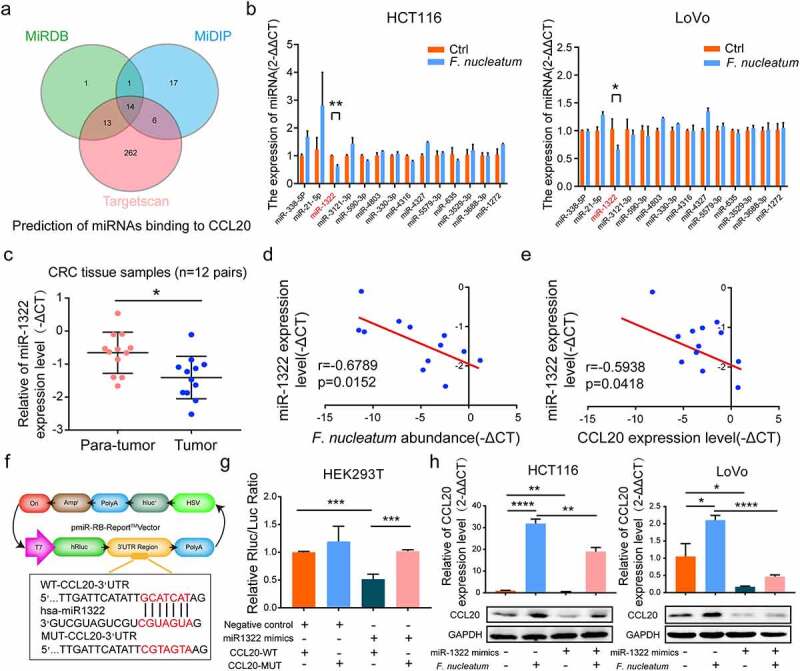
**Tumor-derived CCL20 induced by *F. nucleatum* was mediated by miR-1322**. (a) Public databases were used to predict miRNAs binding to CCL20. Venn diagram showing the overlapping genes. (b) The expression of candidate miRNAs binding to CCL20 were tested in CRC cells after *F. nucleatum* or PBS treatment (Student’s t test). (c) The expression levels of miR-1322 in CRC tissues (n = 12) and the adjacent normal tissues (n = 12, paired Student’s t test) was tested by qPCR. (d, e) The correlation between miR-1322 expression and CCL20 expression or *F. nucleatum* abundance in our tested CRC tissues (n = 12; spearman correlation analysis). (f)Luciferase reporter plasmids which harbor either wild type (WT) or mutant (MUT) miR-1322 binding sites in 3′-URT of CCL20. (g) HEK293T cell was transiently transfected with luciferase constructs with miR-1322 mimics or negative control. After co-cultured 48 h, the luciferase activity was detected (Student’s t test). (h) The protein and mRNA expression of CCL20 were detected in CRC cells by qPCR and Western blot after pretreated with miR-1322 and/or *F. nucleatum* (Student’s t test). * *p* < .05, ** *p* < .01, and *** *p* < .001, **** *p* < .0001, ns no significant

Micro-RNA (miRNA)-mediated gene expression plays an important role in cell homeostasis and carcinogenesis.^20–22^ To determine whether *F. nucleatum* induced CCL20 expression by regulating miRNA, the public prediction databases MiRDB, MiDIP, and Targetscan were used to identify several novel candidate miRNAs (Figure 5(a)). To validate the predicted results and detect the expression of candidate miRNAs, HCT116 and LoVo cells were infected with *F. nucleatum*. We used the qPCR assay to demonstrate that miR-1322 expression was significantly decreased after incubation with *F. nucleatum* in all candidate miRNAs (Figure 5(b)). The expression of miR-1322 was also lower in CRC tissues than in adjacent normal tissues (Figure 5(c)). Moreover, miR-1322 expression was negatively associated with CCL20 expression and *F. nucleatum* abundance in CRC tissues (Figure 5(d-e)). Therefore, we propose that miR-1322 mediates *F. nucleatum*-induced expression of CCL20. To confirm whether miR-1322 directly regulated CCL20 expression, we generated luciferase reporter plasmids harboring either wild-type (WT) or mutated (MUT) miR-1322 binding sites within the 3′-UTR of CCL20 (Figure 5(f)). HEK293T cells were transiently transfected with luciferase constructs along with miR-1322 mimics or negative controls. Consistent with our previous results, miR-1322 mimics significantly suppressed luciferase activity of reporter genes containing the WT compared with MUT 3′-UTR of CCL20 (Figure 5(g)). The miR-1322 mimics downregulated the expression of CCL20 at the protein and mRNA levels, which was upregulated during *F. nucleatum* infection (Figure 5(h)). Taken together, these results suggest that *F. nucleatum* promotes CRC metastasis by regulating the miR-1322/CCL20 axis.

### *F. nucleatum* regulates miR-1322/CCL20 through the NF-KB signaling pathway

**Figure 6.**
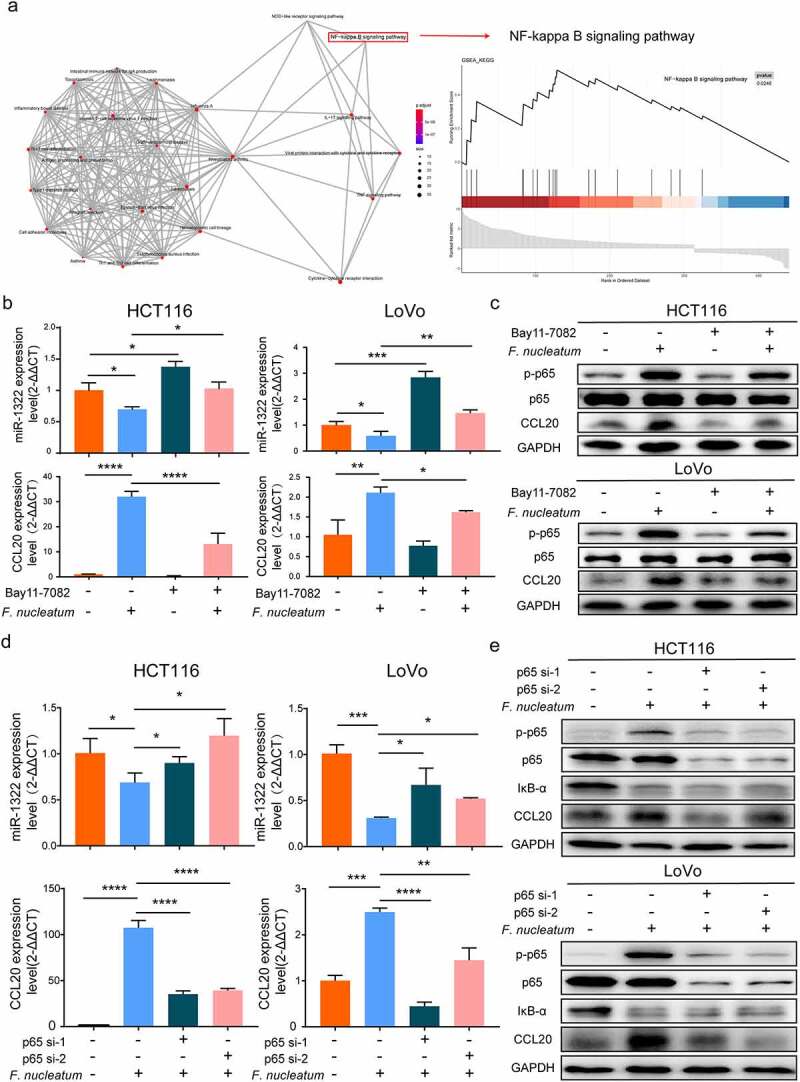
**NF-κB pathway is activated by *F. nucleatum* infection**. (a) KEGG (Kyoto Encyclopedia of Genes and Genomes) and GSEA (Gene Set Enrichment Analysis) analysis were performed based on RNA-seq profiles. The degree of color represented *p*-adjust value and the size of node represented the gene number in this item. (b) After infected with *F. nucleatum* and/or NF-κB pathway inhibitor Bay11-7082 (20 μM). The expression of miR-1322 and CCL20 in CRC cells were tested by qPCR analysis (Student’s t test). (c) After infected with *F. nucleatum* and/or NF-κB pathway inhibitor Bay11-7082 (20 μM), the protein expression of CCL20, phosphorylation level of p65 (*p*-p65), p65 and GAPDH were measured in CRC cells by Western blot. (d) After incubated with *F. nucleatum* and/or p65 siRNA. The expression of miR-1322 and CCL20 were measured by qPCR (Student’s t test). NF-κB subunit p65, *p*-p65, IκB-α, CCL20 and GAPDH were measured by Western blot. Each experiment was repeated in triplicate; * *p* < .05, ** *p* < .01, and *** *p* < .001, **** *p* < .0001

Finally, we examined upstream regulators that might transmit signals to the miR-1322/CCL20 axis. We performed pathway analyses (Kyoto Encyclopedia of Genes [KEGG] and Gene Set Enrichment analysis [GSEA]) based on RNA-seq profiles and found that *F. nucleatum* infection was positively associated with the NF-κB signaling pathway (Figure 6(a)). To verify this result, HCT116 and LoVo cells were treated with the NF-κB pathway inhibitor Bay11-7082 alone or together with *F. nucleatum*. Bay11-7082 increased the miR-1322 expression, and decreased the mRNA expression of CCL20 simultaneously (Figure 6(b-c)). Then we used p65 siRNA to validate this result. The expression of miR-1322 was upregulated by pretreatment with p65 siRNA and *F. nucleatum* in CRC cells, while levels of *p*-p65 and IκB-α were downregulated (Figure 6(d-e)). Together, these results confirm that *F. nucleatum* regulates miR-1322/CCL20 expression through the NF-κB pathway.

**Figure 7.**
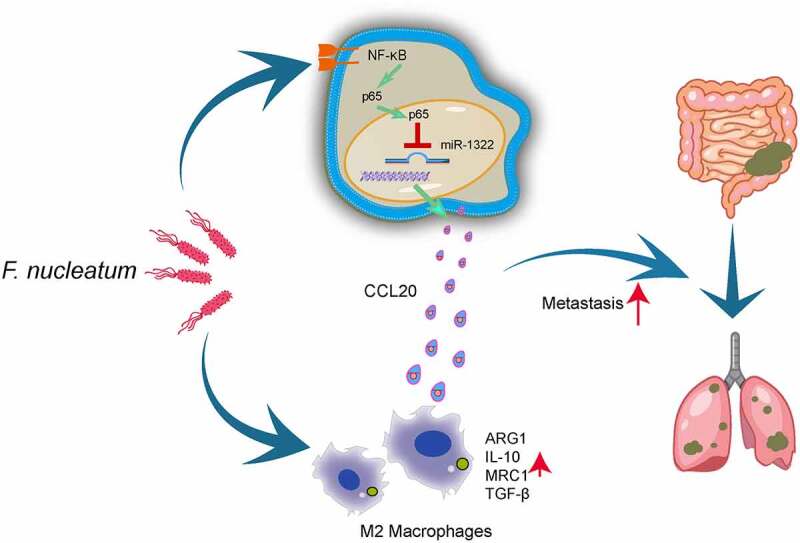
**The illustration of molecular mechanism by which *F. nucleatum* promotes colorectal cancer metastasis**. 1.*F. nucleatum* upregulates CCL20 expression via the NF-κB/ miR-1322 axis, promoting CRC metastasis. 2.*F. nucleatum* promotes macrophage infiltration, simultaneously induces M2 macrophage polarization, enhancing CRC metastasis. 3.Tumor-derived CCL20 expression induced by *F. nucleatum* increased macrophage infiltration in the TME

## Discussion

Although accumulating evidence indicates that *F. nucleatum* abundance is associated with CRC metastasis,^15,23,24^ the association between *F. nucleatum* infection and the host immune system in CRC metastasis has not been determined. In the present study, we found that *F. nucleatum* was highly abundant in the feces and tumor tissues of patients with CRC. Recently, clinical evidence has suggested that the abundance of *F. nucleatum* is higher in feces among metastasis compared with non-metastasis in CRC patients. Although not statistically significant, we found that the abundance of *F. nucleatum* tended to increase in stage IV compared with stage I in patients with metastatic CRC (Figure 1(d)). In the future, we will collect more surgical specimens of stage IV colorectal cancer for further analysis.

To reveal the mechanism by which *F. nucleatum* promotes CRC metastasis, an RNA- seq profiles was developed. CCL20 is one of the most differentially upregulated chemokines associated with CRC, and CCL20 knockdown alleviated the metastasis-promoting function of *F. nucleatum in vivo* and *in vitro*. In addition to the impact of CCL20 on the progression of multiple cancers, it also participates in the remodeling of the TME.^18,25^ Several studies have shown that CCL20 contributes to the progression of cancer by recruiting CCR6^+^ immune cells, such as Treg cells, dendritic cells, Th17 cells, and B cells, to the TME.^26–30^

The intestinal microbiome affects the immune system of the host, and conversely, the immune system adjusts the microbial composition of the gut. *F. nucleatum* plays a vital role in physiological function, especially in regulating the immune system of CRC patients.^31,32^ Recent investigations have reported that *F. nucleatum* binds and activates TIGIT and CEACAM1 expressed by T and NK cells to suppress anti-tumor immunity.^9,33^ Combined with immune checkpoint inhibitors or chemotherapeutics, clearance of *F. nucleatum* decreased immunosuppressive myeloid-derived suppressor cells (MDSCs), remodeled the TME, and prolonged mouse survival.^31^ Additionally, Serna et al. found that *F. nucleatum*-positive, locally advanced rectal cancer patients with nCRT (untreated and post-neoadjuvant) lacked CD8^+^ T cells, leading to high relapse rates.^34^ We used systematic analysis of immune cell infiltration to show that *F. nucleatum* promoted macrophage infiltration and tumor-derived CCL20 in the TME. CCL20 might bridge the communication gap between *F. nucleatum* and macrophage infiltration.

Influenced by specific factors, immune cells were probably transformed, reprogramming the TME to benefit cancer cell survival, ultimately enhancing cancer progression.^35^ Evidence has accumulated that macrophage exhibit various phenotypes, including the M1 or M2 phenotype, to promote tumor rejection or stimulate tumor development, depending on the environment.^36^ Tumor-associated macrophage exhibit mainly an M2-like phenotype and stimulate tumor growth by promoting tumor immune-suppression.^37^ We found that *F. nucleatum* induced M2 macrophage polarization, which promotes the metastasis of CRC. Interestingly, although the expression of M2 macrophage markers was upregulated by *E. coli* (strain DH5α) *in vitro*, no statistical difference was found in AOM/DSS model (Figure 4(a-b)). It is possible that *E. coli* is in direct contact with the macrophage *in vitro*, while the TME is more complicated and closer to reality *in vivo*. Previous evidence has indicated that *E. coli* (strain DH5α) via binding to SR-AI (the class A macrophage scavenger receptor type I) modulates macrophage activation *in vitro*.^38^ Han et al. also found that *E. coli* (strain DH5α) increases TSLP (thymic stromal lymphopoietin) expression in THP-1 and HL-60-derived macrophage, leading to systemic inflammatory reaction and organ dysfunction.^39^ Despite having good immunogenicity, *E. coli* failed to promote CRC tumorigenesis in our AOMD/DSS model, and the results were not statistically significant.

Previous research has shown that miR-1322 can regulate ECRG2 and is a potential diagnostic biomarker for esophageal squamous cell carcinoma.^40^ In addition, Circ-HOMER1 enhances the inhibition of miR-1322 on CXCL6 to regulate the growth and aggressiveness of hepatocellular carcinoma.^41^ We used a public prediction database to confirm that miR-1322 directly regulated CCL20 expression. Our results showed that the expression of miR-1322 was decreased in CRC patients, and *F. nucleatum* regulated the expression of CCL20 via miR-1322. However, the potential role of miR-1322 in CRC remains poorly understood; it will be of great interest to establish the biological importance of miR-1322. NF-κB, an important transcription factor, participates in inflammation-associated CRC. Studies have confirmed that *F. nucleatum* infection activates the NF-κB pathway to regulate miR21/RASA1 in CRC.^23^ Therefore, we tested whether the activated NF-κB pathway was involved in the regulation of miR-1322/CCL20. Through KEGG and GSEA analyses based on our RNA-seq profiles, we found that *F. nucleatum* regulated miR-1322/CCL20 through the NF-κB pathway.

In conclusion, we confirmed a novel association between *F. nucleatum* and macrophage infiltration and elucidated the molecular mechanisms underlying the regulation of macrophage in CRC metastasis (Figure 7). Our data offer a new opportunity for targeting chemokines and immune homeostasis for the treatment of CRC metastasis.

## Materials and methods

### GMrepo database analysis

The GMrepo is a curated and annotated human gut metagenomic data repository for microbial abundance information at the species level. The *F. nucleatum* relative abundances among the stool samples of the healthy and CRC patients from GMrepo was obtained with GMrepo RESTful APIs for R (version 3.6.1 https://www.r-project.org) and RStudio (version 1.1.442 https://www.rstudio.com) software. The data quality was assessed by consulting the data of publications. Then, the relative abundance of *F. nucleatum* among the healthy and CRC patients were extracted.

### Clinical samples

Published data of 489 colorectal carcer and 2411 normal tissues from ATCC (The Cancer Genome Atlas) were used to analyze *F. nucleatum* abundance. Stool samples were obtained from 63 patients of CRC and 59 healthy people for physical examination from Sir Run Run Shaw Hospital, School of Medicine, Zhejiang University (cohort 1). All 77 pairs colorectal carcinomas and adjacent normal tissues (cohort 2) were collected from patients, and written informed consent before collection were obtained in Sir Run Run Shaw Hospital (Zhejiang, China). The protocol was agreed by the Clinical Research Ethics Committee of the Sir Run Shaw Hospital, School of Medicine at Zhejiang University.

### Bacterial strain and cell culture

*F. nucleatum* (ATCC 25586) was purchased from American type culture collection and grown in Columbia blood agar under an anaerobic jar (MITSUBISHI Gas Chemical Co., Japan) at 37°C. *E. coli* strain DH5α (Takara, Japan) was cultured in Luria-Bertani medium. HCT116 and LoVo (CRC cell lines) were purchased from ATCC. HCT116 was maintained in Maccoy 5A medium (Genom, China) and LoVo was cultured in F-12 K medium (Genom, China). The medium contained 10% FBS (fetal bovine serum) and 1% Peni-cillin/Streptomycin. All CRC cells were cultured in a 5% CO_2_-humidified incubator.

### DNA extraction and bacteria quantification

Bacteria DNA from fecal samples were extracted with QIAGEN stool kits (QIAGEN, Germany). *F. nucleatum* quantification was performed by quantitative real-time PCR. Each reaction was tested in triplicate including SYBR Premix Ex Taq (Takara, Japan), primers and template DNA. Relative abundance was calculated by -ΔCt method. Universal Eubacteria 16s was used as internal reference gene. The primer sets used were:

*F. nucleatum* -F, 5ʹ-CGGGTGAGTAACGCGTAAAG-3ʹ,

*F. nucleatum* -R, 5ʹ-ACATTGTGCCACGGACATCTTG −3ʹ;

universal Eubacteria 16s-F, 5ʹ-CGGCAACGAGCGCAACCC-3ʹ,

universal Eubacteria 16s-R, 5ʹ-CCATTGTAGCACGTGTGTAGCC-3ʹ

### Wound healing assays

The back of 6-well plate was marked with horizontal lines evenly, and then CRC cells were seeded overnight. 200ul pipette tips were used to wound in the 6-well plate and then cells were infected with *F. nucleatum* (MOI: multiplicity of infection = 100:1) or *E. coli* (MOI = 1:50). Images of cells were observed at 0, 24 h, 48 h, and 72 h, respectively.

### Transwell migration assays

Cell migration was performed in Corning transwell insert chambers (8.0 μm pore size) according to the manufacturer’s instructions. CRC cells with serum-free medium were seeded in the upper well of Corning transwell insert chamber (8.0 μm pore size), medium (500 μl) with 10% FBS or different treated supernatant of macrophage medium were added to the lower chambers for 24 h. For CCL20 mediated the macrophage infiltration assay, macrophage with serum-free medium were seeded in the upper well of Corning transwell insert chamber, culture medium of CRC cells pretreated by *F. nucleatum* alone or together with si-RNA CCL20 for 24 h, was added to the lower chambers. Then the transwell insert chamber membrane was fixed with 4% paraformaldehyde for 15–20 min and stained with crystal violet for 15 min. Finally, images of cells were observed and randomly taken under fluorescence microscope. Every 6 fields were counted for each sample.

### Western blotting

CRC cells were seeded in 6-well plate and treated with different experimental methods. Proteins were extracted via RIPA lysis buffer (Solarbio, China) and centrifuged to remove the insoluble components. The supernatant was run on different density SDS-PAGE and then transferred to a PVDF mem-brane. After being blocked with Blocking Buffer (Beyotime, China) for 15 min, the membrane was incubated with CCL20 (A1756, ABclonal), *p*-p65 (3033S, Cell Signaling Technology), p65 (8242S, Cell Signaling Technology), IκB-α (4814S, Cell Signaling Technology), GAPDH (A19056, ABclonal) antibody overnight at 4°C and subsequently incubated with second HRP-linked antibody. ECL detection kit was used to detect the blots according to the manufacturer’s procedure.

### RNA sequencing

RNA sequencing was performed as described previously.^15^ Briefly, after incubated with F. nucleatum (MOI = 100:1) or PBS for 24 h, total RNAs of LoVo cells were extracted and subjected to cDNA synthesis followed by adaptor ligation and enrichment. The RNA sequencing was paired-end sequenced at Guangzhou RiboBio Co., Ltd. (China). The whole samples expression levels were presented as RPKM (Reads Per Kilobase per Million). All differentially expressed genes were ploted with “pheatmap” and “ggplot2” R packages. For KEGG and GSEA enrichment analysis, a *p* value <.05 was used as the threshold to determine the significant enrichment of the gene sets with “clusterProfiler”R package. For immune cells infiltration estimations, the whole samples expression levels were presented TPKM (Transcripts Per Kilobase Million). The RNA sequencing dataset has been deposited in the Gene Expression Omnibus (GEO) accession: GSE173549.

### RNA extraction and quantitative real-time PCR

Total RNAs of cell lines (HCT116 and LoVo) and tissue were extracted by TRIzol reagent (Takara, Japan) and then was reversely transcribed as cDNA via PrimeScript™ RT reagent Kit (Takara, Japan). Total miRNAs of cell lines (HCT116 and LoVo), which incubated with *F. nucleatum* (MOI = 100:1) or PBS for 24 h, were extracted by TRIzol reagent (Takara, Japan) and then was Mir-X^TM^ miRNA First-Strand Synthesis Kit (Cat#638313 Takara, Japan) according to manufacturer’s instructions. Quantitive real-time PCR was performed using SYBR Premix Ex Taq (Yeasen, China) in the Light Cycler®480 Real-Time PCR System (Roche). Each reaction was tested in triplicate. U6 or GAPDH was used as the internal reference and the 2(-ΔΔCt) method was used for calculating the relative mRNA or miRNA expression.

The following primer sets were used:

Mouse CCL20: Forward: 5ʹ- ACTGTTGCCTCTCGTACATACA-3ʹ,

Reverse: 5ʹ- GAGGAGGTTCACAGCCCTTTT-3ʹ;

Human CCL20: Forward: 5ʹ- TGCTGTACCAAGAGTTTGCTC-3ʹ,

Reverse: 5ʹ- CGCACACAGACAACTTTTTCTTT-3ʹ;

Human TGF-β: Forward: 5ʹ- CTAATGGTGGAAACCCACAACG-3ʹ,

Reverse: 5ʹ- TATCGCCAGGAATTGTTGCTG-3ʹ;

Mouse TGF-β: Forward: 5ʹ- GAGCCCGAAGCGGACTACTA-3ʹ,

Reverse: 5ʹ- TGGTTTTCTCATAGATGGCGTTG-3ʹ;

Human ARG-1: Forward: 5ʹ- GTGGAAACTTGCATGGACAAC-3ʹ,

Reverse: 5ʹ- AATCCTGGCACATCGGGAATC-3ʹ;

Mouse ARG-1: Forward: 5ʹ- CTCCAAGCCAAAGTCCTTAGAG-3ʹ,

Reverse: 5ʹ- GGAGCTGTCATTAGGGACATCA-3ʹ;

Human IL-10: Forward: 5ʹ- TCAAGGCGCATGTGAACTCC-3ʹ,

Reverse: 5ʹ- GATGTCAAACTCACTCATGGCT-3ʹ;

Mouse IL-10: Forward: 5ʹ- CTTACTGACTGGCATGAGGATCA-3ʹ,

Reverse: 5ʹ- GCAGCTCTAGGAGCATGTGG-3ʹ;

Human MRC-1: Forward: 5ʹ- GGGTTGCTATCACTCTCTATGC-3ʹ,

Reverse: 5ʹ- GCCTGATGCCAGGTTAAAGCA-3ʹ;

Mouse MRC-1: Forward: 5ʹ- GAGGGAAGCGAGAGATTATGGA-3ʹ,

Reverse: 5ʹ- GCCTGATGCCAGGTTAAAGCA-3ʹ;

Human miR-1322: Forward: 5ʹ- CGATGATGCTGCTGCTGCTG −3ʹ.

### Cell transfection

MiR-1322 mimic, siRNA-CCL20, knock-down plasmid (pcDNA3.1-shCCL20) and its negative control were obtained from RiboBio (Guangzhou, China). Cell transfection was accomplished using Lipofectamine™ RNAiMAX (Thermo Fisher Scientific, Massachusetts, USA) or FuGENE HD transfection reagent (Promega, USA) in opti-MEM according to the provided protocols.

### Bioinformatics analysis

The miRNAs binding to CCL20 were predicted by three bioinformatics databases: Targetscan (http://www.targetscan.org/), MirDIP (http://ophid.utoronto.ca/mirDIP) and MiRDB (http://mirdb.org/). GSEA and KEGG pathway enrichment analysis was conducted by “clusterProfiler” package (version 3.5) in R (https://www.r-project.org,version 3.6.0).

### Luciferase reporter assay

Wild-type (wt) and mutant (mut) 3ʹ-UTR of CCL20 luciferase reporter plasmid vectors were obtained from RiboBio (Guangzhou, China). The reporter plasmid was co-transfected with miR-1322 mimics or negative control miRNA into HEK293T. Dual-Luciferase Reporter Assay System (Yeasen, China) was used to detect the luciferase activity after 48-hour incubation.

### Induction of pulmonary metastasis model and AOM/DSS CRC model

For pulmonary metastasis model, the five-week-old BALB/c nude mice were purchased from Shanghai SLAC Laboratory Animal Co. China. After pretreated with *F. nucleatum* (MOI = 100:1) or PBS for 24 h, control shRNA or CCL20 shRNA HCT116 cells were injected via tail vein. Under the normal fed condition for two months in specific pathogen-free conditions, the mice were sacrificed. Then the lungs of mice were removed, photographed, and measured. Subsequently, lung metastatic lesions were fixed with neutral formalin and embedded with paraffin. Representative metastasis lungs tumor and H&E (hematoxylin and eosin) staining images were presented. For AOM/DSS model, five-week-old male C57/B6 mice were divided into three groups (PBS group, *F. nucleatum* group and *E. coli* group), and injected with 10 mg/kg AOM (Sigma), After 5 days, 2.5% DSS was given in water for 5 days, then by regular drinking water for 2 weeks. Finally repeating this cycle twice, mice were sacrificed. Mice in group *F. nucleatum* was administrated 1 × 10^9^ CFU (colony-forming units), *E. coli* group was administrated 5 × 10^8^ CFU, while PBS alone as control group. *F. nucleatum* or *E. coli* was transplanted every 2 days. Colon tissues were removed and collected for Flow cytometry analysis. All methods were carried out in accordance with guidelines and regulations of the Animal Experimentation Ethics Committee at Zhejiang University.

### Generation and polarization of macrophage

To generate macrophage, bone marrow-derived macrophages (BMDMs) were collected by flushing the femur and tibia of BALB/c mice. These BMDMs were cultured in DMEM medium (Genom, China) contained 10% FBS and 5% M-CSF (Macrophage Colony stimulating Factor; Novoprotein, China) about 1 week. BMDMs were confirmed by flow cytometry for F4/80. THP-1 cell line was purchased from American type culture collection (ATCC) and cultured in RPMI-1640 medium contained 10% FBS and 200 nM PMA (phorbol-12-myristate-13-acetate, Sigma) for 48–72 h.To induce polarization of macrophage, BMDMs and THP-1 were collected and treated with *F. nucleatum* (MOI = 100:1) or *E. coli* f(MOI = 50:1) for 24 hours.

### Immunofluorescence

Paraffin embedded sections (5 μm) of colorectal cancer tissue (*F. nucleatum* high abundance and *F. nucleatum* low abdundance) were performed. After blocked with 5% BSA, Paraffin embedded sections were stained by F4/80 (GB11027, Servicebio, China), CCL20 (26527-1-AP, Proteintech, China), CCR6 (GB11027, Servicebio, China), CD68 (GB11027, Servicebio, China), CD206 (GB11027, Servicebio, China) antibody and DAPI (Servicebio, China). All pictures were analyzed by light microscopy.

### Flow cytometry

Cells from colon lamina propria were collected and then incubated cell surface florescence-conjugated antibodies (BV421-F4/80: 565411, BD Biosciences; AF488-CD11b: 557672, BD Biosciences; CD206 (560408, BD Biosciences); Alexa Fluor 700-CD45: (560510, BD Biosciences) at room temperature for 30 min. Subsequently, cell samples were analyzed using Flow Cytometer (BD Biosciences) and the FlowJo software (Tree Star Inc., San Carlos, CA) was performed to analyze the results.

### Immune cells infiltration estimations by TIMER 2.0 and quanTIseq

TIMER 2.0(http://timer.cistrome.org/) is a comprehensive resource for systematical analysis of immune cells infiltrate across diverse cancer types. QuanTIseq is an immune deconvolution method to estimate immune infiltration. RNA-seq profiles was used, and the entire samples expression levels were presented as TPM (Transcripts Per Kilobase Million). After RNA-seq profiles input file is uploaded, the estimation component of TIMER 2.0 will automatically run immune infiltration estimation by multiple immune deconvolution methods, including quanTIseq, TIMER, CIBERSORT, xCell, MCP-counter and EPIC algorithms. The estimated values of all immune cell infiltration, eight common immune cells infiltration level and the proportion of common immune cells infiltration of each sample are showed by table or images.

### Statistical analysis

Statistical analysis was performed using GraphPad Prism (GraphPad Software, San Diego, CA, USA). The one-way ANOVA, paired or unpaired Student’s t test, Mann Whitney test or spearman correlation analysis was used to analyze. Data were expressed as mean ± Standard Deviation (SD) or Standard Error of Mean (SEM). *P* value less than 0.05 was considered statistically significant. The significance was defined as * *p* < .05, ** *p* < .01, *** *p* < .001, and **** *p* < .0001.

## Data Availability

The RNA sequencing data has been deposited to the Gene Expression Omnibus (GEO) under the accession number GSE173549. https://www.ncbi.nlm.nih.gov/geo/query/acc.cgi?acc=GSE173549.
